# Effects of Hypergravity on Phase Evolution, Synthesis, Structures, and Properties of Materials: A Review

**DOI:** 10.3390/ma18030496

**Published:** 2025-01-22

**Authors:** Yisheng Zheng, Lilin Xie, Yanhui Chen, Xiaodong Han

**Affiliations:** 1 Institute of Microstructure and Property of Advanced Materials, Beijing University of Technology, Beijing 100124, China; 2 Institute of Quantum Materials and Physics, Henan Academy of Sciences, Zhengzhou 450046, China

**Keywords:** hypergravity, material synthesis, phase separation, structural evolution, mechanical properties

## Abstract

In a hypergravity environment, the complex stress conditions and the change in gravity field intensity will significantly affect the interaction force inside solid- and liquid-phase materials. In particular, the driving force for the relative motion of the phase material, the interphase contact interaction, and the stress gradient are enhanced, which creates a nonlinear effect on the movement mode of the phase material, resulting in a change in the material’s behavior. These changes include increased stress and contact interactions; accelerated phase separation; changes in stress distribution; shear force and phase interface renewal; enhanced interphase mass transfer and molecular mixing; and increased volume mass transfer and heat transfer coefficients. These phenomena have significant effects on the synthesis, structural evolution, and properties of materials in different phases. In this paper, the basic concepts of hypergravity and the general rules of the effects of hypergravity on the synthesis, microstructure evolution, and properties of materials are reviewed. Based on the development of hypergravity equipment and characterization methods, this review is expected to broaden the theoretical framework of material synthesis and mechanical property control under hypergravity. It provides theoretical reference for the development of high-performance materials under extreme conditions, as well as new insights and methods for research and application in related fields.

## 1. Introduction

When a material is on the Earth’s surface, it is subjected to the force of gravity, resulting in a normal-gravity environment with Earth’s average gravitational acceleration, *g*, of 9.81 m/s^2^. However, when the acceleration exceeds the temporal gravitational acceleration, the force acting on the material is known as hypergravity, resulting in a hypergravity environment. Hypergravity conditions are widespread in the environments in which people live, such as in matter under conditions of centrifugal motion, where the centrifugal acceleration can be much greater than the gravitational acceleration. Hypergravity environments can be achieved through centrifugal force generated by rotation [1], which includes equipment such as centrifuges, rotating packed beds, and turbines. In centrifugal motion, materials experience centrifugal force, interphase interaction forces, and the Coriolis force. Compared to normal gravity, hypergravity enhances the self-weight stress of multiphase media and increases its gradient. It also increases the driving force for relative motion between different phases by enhancing interphase interaction forces. Therefore, the fluid flow properties and interphase interaction in multiphase systems under hypergravity conditions play a crucial role in the phase evolution of materials. The phase evolution in multiphase materials under hypergravity conditions will be different from that in materials under conventional gravity conditions. For example, under hypergravity conditions, the solidification of alloy melts leads to grain refinement and the weakening of dendrites [2,3], changes in boundary layer structure and flow characteristics in fluids [4,5], strengthening reactions and the synthesis of nanomaterials [6,7], and the separation of two-phase materials in metallurgical processes [8,9].

Hypergravity is also widely present in components with a single solid phase, such as turbines used in gas turbines and engines in industries. The study of the structural evolution of solid materials under hypergravity conditions is of crucial importance for industrial safety. However, in the study of solid materials under hypergravity conditions, the effects of hypergravity are often overlooked. The effects of a hypergravity environment on components such as turbines is often accompanied by high temperatures and stresses [10]. The extreme conditions of high temperature and complex stress have often led previous studied to simplify hypergravity to conventional stress, focusing more on the effects of high-temperature and high-stress coupling on solid materials while neglecting the effects of stress gradients and interphase interaction forces caused by hypergravity. Taking superalloys under high-temperature hypergravity conditions as an example, previous studies have used conventional stress applied under normal gravity to simulate the effects of hypergravity on the structural evolution, mechanical behavior, and properties of materials. This simplification has led to discrepancies in creep between simulated rafting structures and those in service alloys, as well as lower simulated creep life compared to actual service life. This phenomenon may be related to the directional diffusion of Ni/Al atoms caused by centrifugal stress gradients under hypergravity, which determines the dependence of precipitate morphology evolution in Ni-based alloys [11]. Therefore, the enhanced mass transfer under hypergravity and its impact on other characteristics significantly affect the microstructure and behavior of materials, indicating that the influence of hypergravity on the diffusion, microstructural evolution, and mechanical behavior of solid alloy materials cannot be simplified or ignored. 

Element diffusion is the basis of structural evolution in solid materials, which in turn affects the microstructural evolution and mechanical behavior and properties of materials. Therefore, studying the diffusion laws and mechanisms of elements under hypergravity conditions and elucidating the physical mechanisms of hypergravity’s effect on solid-state diffusion and phase evolution are fundamental for studying the mechanical behavior and properties of solid materials under hypergravity. This will lead to a deeper understanding of the mechanical behavior of solid materials under hypergravity environments. The aforementioned research provides possibilities for revealing the diffusion laws and mechanisms, mechanical behavior, and other aspects of solid materials under hypergravity conditions. It also provides a theoretical foundation and experimental basis for the development and failure analysis of high-performance alloy materials under high-temperature hypergravity conditions. Furthermore, it has important practical and scientific implications for the application of solid materials in complex hypergravity environments.

The architecture of the paper is organized as follows: Section 1 introduces the background of hypergravity research; Section 2 elaborates on the principles and techniques of hypergravity; Section 3 discusses the liquid-phase synthesis and separation of materials under hypergravity conditions; Section 4 presents the metallurgy of alloys under hypergravity conditions; Section 5 analyzes the structure and properties of alloy materials under hypergravity; and Section 6 concludes with outlooks and future prospects. 

In this review, we adopted a systematic approach to collect and analyze the corresponding literature on hypergravity-related research fields, such as the fundamental concept of hypergravity, hypergravity technologies, phase evolution, materials preparation, phase separation, centrifugal casting, and properties. We conducted a comprehensive review of the literature to identify studies directly related to the effects of hypergravity on materials-related multidisciplinarity. It is designed to provide researchers, engineers, and students of disciplines in the field of materials science related to hypergravity with a comprehensive perspective to inform and inspire their research and practice. 

## 2. Principles and Techniques of Hypergravity

### 2.1. Hypergravity Condition

High-speed rotation of a centrifuge is an effective method to create a stable hypergravity field. For centrifugal hypergravity environments, the origin of the stationary coordinate system and the moving coordinate system are respectively located at the axis of the centrifuge and the center of the moving object (such as the experimental chamber). The governing equation for a moving object of uniform mass, m, in the rotating coordinate system, a hypergravity condition with a constant rotational speed, can be described with Equation (1) [12].

(1) $$\frac{md_{r}^{2}r}{dt^{2}}=F-m\omega \times (\omega \times R)-m\omega \times (m\omega \times r)-m\omega \times \nu^{\prime }$$

where ***F*** is the external force, ***ω*** is the rotational angular velocity vector, ***R*** is the rotation radius vector of the stationary coordinate system, ***r*** is the position vector of the moving coordinate system of particle (sample in the experimental chamber), and ***v***′ is the velocity vector of the sample relative to the moving coordinate system. The second term on the right-hand side of the equation, mω×(ω×R), represents the linear hypergravity (centrifugal) force; the third term, mω×(ω×r), represents the nonlinear centrifugal force; and the last term represents the Coriolis force. 

The hypergravity coefficient, *G*, which describes the magnitude of the hypergravity field, is defined as the ratio of centrifugal acceleration to normal gravitational acceleration. It is defined according to Equation (2):

(2) $$G=\frac{\omega^{2}R}{g}=\frac{N_{r}^{2}\pi^{2}R}{900g},$$

where *N_r_* is the rotation speed, and the hypergravity intensity could be denoted as *Ng*, where *N* is an integer greater than 1. The unit of hypergravity coincides with the unit of gravitational acceleration, i.e., m/s^2^.

According to Equation (1), the hypergravity acting on samples can be categorized into two main components: the centrifugal force induced by rotation, *F_g_*, and the Coriolis force resulting from variations in movement relative to the coordinate system during rotation, *F_c_*. However, for materials that are not of a single composition, there will be a buoyancy force, *F_b_*, due to variations in the species density, which is one of the interphase forces. Therefore, the above three forces and their effects will be mainly discussed in this review.

The centrifugal force, which is the most significant force acting on the sample, plays a crucial role in the diffusion and structural evolution processes of materials. It can be described as follows:

(3) $${dF}_{g}=S\rho \omega^{2}RdR,$$

where *S* is the cross-sectional area, and *ρ* is the density of the sample. Therefore, according to Equation (3), the stress, *σ*, induced by the centrifugal force can be described as:

(4) $$\sigma =\int_{R_{1}}^{R_{2}}\rho \omega^{2}RdR=\frac{1}{2}\rho \omega^{2}\left(R_{2}^{2}-R_{1}^{2}\right),$$

where *R*_1_ and *R*_2_ are the distances of the sample edges from the center of rotation. 

*F_b_* can be described as:

(5) $$F_{b}=∆m\omega^{2}R,$$

where ∆*m* is the mass difference of the species, which originates from the density differences in phases, grains, or atoms based on analytical scales (∆*m* is relevant to ∆*ρV*, where *V* is the volume). Obviously, *F_b_* increases with the increasing hypergravity according to Equation (5). 

The Coriolis force is described in the following:

(6) $$F_{c}=-2m\omega \times \nu^{\prime },$$

It should be noted that both ***ω*** and ***v*** in this context are vectors, so the magnitude of *F_c_* is related to the cross product of ***ω*** and ***v***′.

Obviously, both *F_g_* and *F_b_* are significant for the multiphase system, in accordance with Equations (3)–(5). For the liquid-phase system, the role of *F_c_* cannot be neglected, but for the solid-phase system, *F_c_* can be neglected because ***v***′ is relatively small, according to Equation (6).

### 2.2. Enhanced Interphase Interaction and Mass Transfer Under Hypergravity Conditions

**Figure 1:**
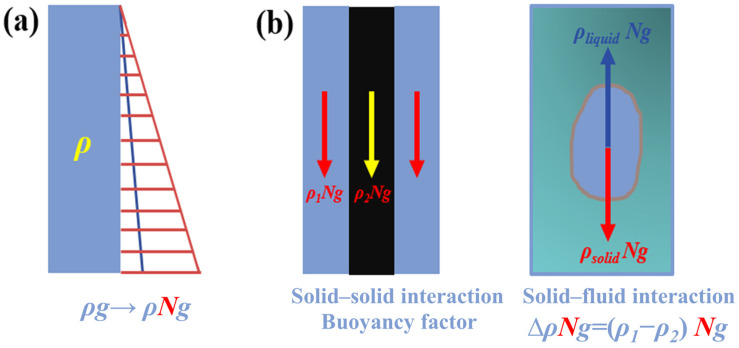
Effects of hypergravity on the interaction forces of matters [1]. (**a**) Increasing the force and gradient; (**b**) strengthening the relative motion between phases.

Equations (1)–(7) describe the stress condition of the sample in hypergravity; most notably, there is an increase in the coefficient of hypergravity and a significant increase in the force on the matter [1], as shown in Figure 1a. Moreover, the buoyancy factor increases between two phases of matter where there is a difference in density, and the interaction force is enhanced, thus enhancing the relative motion between the two phases, as shown in Figure 1b, which shows two solid-phase media in contact with densities of *ρ*_1_ and *ρ*_2_. Under the action of gravity, the relative motion is the driving force (*ρ*_1_ − *ρ*_2_)*g* at the interface of the media. Hypergravity increases the gravitational acceleration, thereby increasing the driving force of the relative motion to (*ρ*_1_ − *ρ*_2_)*Ng*. Likewise, as the buoyancy factor rises under hypergravity, the driving force of the relative motion of the solid–liquid two-phase medium interface is also enhanced.

On the other hand, the enhanced interphase interaction and mass transfer under hypergravity are also reflected in the enhancement of fluid motion. A higher coefficient of hypergravity also means that the linear velocity of the fluid/matter is faster, so the contact between the two phases is more rapid. The hypergravity environment enhances natural convection due to the concentration, thermal gradients, buoyancy, and hydrostatic pressure, which also promotes interphase interaction and mass transfer. Additionally, the domain in the flat solid–liquid interface is also modulated by the hypergravity condition, since the increasing acceleration/gravity decreases the domain height and increases the interfacial free energy [13]. This will significantly affect the solid–liquid interface structure and promote the interaction between the two phases. It should be noted that the phase components exhibit a completely different motion pattern under hypergravity than normal gravity, resulting in a nonlinear transformation of the material motion pattern, also known as nonlinear effects. This means that under the action of external conditions (such as electric fields, magnetic fields, and a series of external physical fields), the response of the material does not follow a simple linear relationship but shows more-complex nonlinear characteristics [1].

The diffusion mass transfer rate between substances is related to the diffusion coefficient, *D*, and the diffusion layer thickness, *δ*, expressed as *D/δ*. In multiphase media, hypergravity enhances phase contact and mixing and increases the diffusion coefficient [14]. Previous studies have shown that the diffusion layer thickness is inversely proportional to the gravity coefficient in a logarithmic manner [15,16], which reduces the boundary layer thickness, thus significantly promoting mass transfer processes.

The enhanced interphase interaction and mass transfer present in hypergravity environments are interrelated and closely associated with the significant acceleration of each phase under hypergravity. Additionally, the buoyancy factor between phases increases with the augmentation of hypergravity. Moreover, flow characteristics such as turbulence and enhanced shear forces in a hypergravity environment also contribute significantly to interphase interaction and mass transfer.

It is important to note that hypergravity does not affect the chemical equilibrium of the system, which is why conventional thermodynamics do not consider the gravitational field. While hypergravity increases the microscopic chemical potential of substances, since factors such as buoyancy and stress gradient in hypergravity change the distribution of compositions, and the microscopic chemical equilibrium remains unchanged, the distribution of solutes in a solution theoretically changes. However, due to the much stronger intermolecular and interionic forces compared to the effects of hypergravity, the changes in solute distribution are minimal, only becoming significant when the hypergravity coefficient and the height difference of hypergravity direction are substantial. Therefore, solely relying on hypergravity to affect the chemical potential of substances for the enrichment or separation of solutes in a solution or components in a melt is difficult to achieve.

Nevertheless, when there is a concentration gradient of solutes in solution and material melt, hypergravity can accelerate solute transfer by enhancing natural convection. Additionally, when there is a density difference between multiphase media, the effect of hypergravity buoyancy becomes significant. The buoyancy factor, Δ*ρNg*, is directly proportional to the gravity coefficient, as shown in Equation (6). Therefore, hypergravity can accelerate phase separation, achieving the separation of phases with different densities, even when the density difference between the two phases is small, such as in solid–liquid phase separation.

Furthermore, since hypergravity is directional, it is an effective method for the deep separation of fine, dispersed phases in a melt. The directionality of hypergravity is reflected in the directionality of the hypergravity buoyancy force, which affects the relative motion of phases/elements with different masses (relatively light phases/elements/defects move in the direction of the small hypergravity force), thereby affecting the flow properties of the fluid material, as well as the diffusion and elemental distributions of the solid-phase material, resulting in structural and property differences. A more in-depth discussion of the effects of hypergravity on fluids will be further described in the subsequent sections.

### 2.3. Hypergravity Techniques

High-speed rotational motion is an effective method to create a stable hypergravity field. Technologies based on centrifugal hypergravity environments can be divided into two categories, i.e., equipment applied to liquid and solid phases, respectively. The former is represented by the rotating packed bed (RPB) and the latter by centrifuge-based equipment. Here, we briefly summarize these two types of techniques; relevant details can be found in some of the previous review articles of RPB [17,18,19] and centrifuge-based techniques.

#### 2.3.1. Rotating Packed Bed Techniques

**Figure 2:**
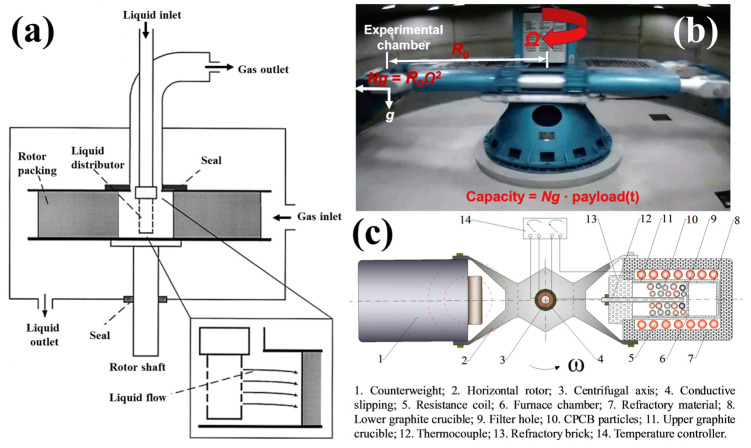
Centrifugal hypergravity equipment. (**a**) Diagram of the rotating packed bed [38]; (**b**) the beam centrifuge [1]; (**c**) the centrifugal hypergravity apparatus [39].

The RPB technique was proposed by Dr. Ramshaw in 1979 [20], in which the hypergravity technology is based on a rotating packed bed (RPB) as the core device, as shown in Figure 2a. The rotating packed bed generates a centrifugal force field through the high-speed rotation of internal rotating components, typically achieving gravity accelerations of hundreds to thousands of times; the technique is also known as hypergravity (Higee) [21,22,23]. 

The RPB is a tool consisting of a rotating part (i.e., an annular rotor with a bed of packing material) and a static part (i.e., the housing). The rotor and the static part, i.e., the housing, are connected by bearings and seals. The rotor is driven by a motor, and the hollow part of the rotor is referred to as the “eye”, which can be identified by an eye ring and contains the liquid distribution. The rotational speed can be adjusted according to process requirements, and the high-speed rotational motion of the packing bed generates a hypergravity environment. The liquid flows into the eye of the rotor, impacts the rotating packing, flows through the packing in the direction of the centrifugal force applied, then flows outward, along the centrifugal force direction towards the outer edge, and is then sprayed into the housing, exiting the entire device through baffle plates. In a classical rotating packed bed, the porous packing material is generally placed inside the rotating components. Under the strong centrifugal force field, the liquid phase collides with the rapidly rotating packing material, resulting in shearing to form liquid films, liquid lines, and liquid droplets, thus increasing the gas–liquid contact area, enhancing the phase interface renewal rate, and intensifying mass transfer between phases. Compared to traditional tower equipment, the gas–liquid mass transfer coefficient in a rotating packed bed can be increased by one to three orders of magnitude [24]. Hypergravity technology greatly enhances gas–liquid mass transfer processes and liquid–liquid mixing processes; significantly reduces equipment size; lowers investment costs; improves intrinsic safety; and promotes quality improvement, efficiency enhancement, energy conservation, and carbon reduction in the chemical industry. Therefore, it is widely applied in processes such as acid gas removal [25,26,27,28], chemical manufacturing [29], alkali liquor oxidation regeneration [30], continuous distillation [31], hydrogenation [32], the preparation of nanoparticles [33], and so on.

In the classical rotating packed bed, the gas phase serves as the continuous phase, while the liquid phase acts as the dispersed phase. The dispersion and size of the liquid droplets significantly affect the performance of the rotating packed bed. Therefore, extensive experimental [34,35] and computational fluid dynamics studies [36,37] have been conducted on the interaction processes between the liquid phase and surface micro-/nano-structured packing materials.

#### 2.3.2. Centrifuge Techniques

Centrifuge techniques involve the sample being placed in the sample compartment of the centrifuge, as shown in Figure 2b,c, rather than the liquid sample filling the entire rotating part, as in the case of the RPB techniques. By installing different onboard equipment on the centrifuge, an experimental platform is provided to elucidate the material transport mechanisms and phase interactions of multiphase media. Interdisciplinary centrifugal hypergravity experiments have been conducted in the fields of materials science and life sciences. Large centrifuges are commonly used in geotechnical engineering related hypergravity environments, with a capacity of 400 g·t [40], as shown in Figure 2b. Large-capacity centrifuges can be used for simulating the dynamic response of rock and soil infrastructure under seismic or explosive events, as well as for studying the long-term migration of pollutants, simulating dam behavior under earthquakes [41], the accumulation-compaction process of mid-ocean ridge minerals, calculating magma migration rates in the region [42], and researching the effects of gravity in life sciences [43].

While small-capacity centrifuges are used in solid-phase materials science for hypergravity research, as shown in Figure 2c, this is the main tool used in current research studies on materials science performed in hypergravity environments, which we will discuss in detail in this paper. The centrifugal motion of the loading chamber in the centrifuge creates a hypergravity environment for the samples contained within it, allowing for targeted scientific experiments by applying different conditions to the sample chamber. For example, common practices include resistive heating of the sample chamber to conduct high-temperature hypergravity experiments. The structure and materials of the reactor can be designed accordingly based on the experimental objectives. For example, solid samples can be used to study solid-phase diffusion and structural evolution experiments; crucibles can be used for sedimentation, solidification, separation, and enrichment experiments of different phases; and crucibles with microporous filtration plates can be used for thermal filtration and separation experiments of solid and liquid phases.

## 3. Liquid-Phase Synthesis and Separation of Materials Under Hypergravity

### 3.1. The Fluid Dynamics Under Hypergravity 

In normal gravity, fluid convection is an important phenomenon that describes the motion of fluids under external forces. Turbulence is an irregular motion state in fluids, characterized by the irregular distribution of velocity and pressure [44]. Vorticity is a rotational motion in fluids, commonly observed in atmospheric and oceanic circulations. Additionally, intermolecular forces in liquids are important phenomena in fluids, including surface tension and viscosity. These phenomena interact with each other; for example, turbulence may lead to the formation of vorticity, and intermolecular forces can affect the motion state of fluids. Finally, the influence of external force fields on various phenomena in fluids is also a key focus of research. External force fields can alter the flow patterns and motion states of fluids, thereby affecting the behavior of fluid dynamics. The various phenomena and interactions in fluids and fluid dynamics constitute a complex and intriguing research field that holds significant importance for understanding fluid motion and phenomena in the natural world. 

**Figure 3:**
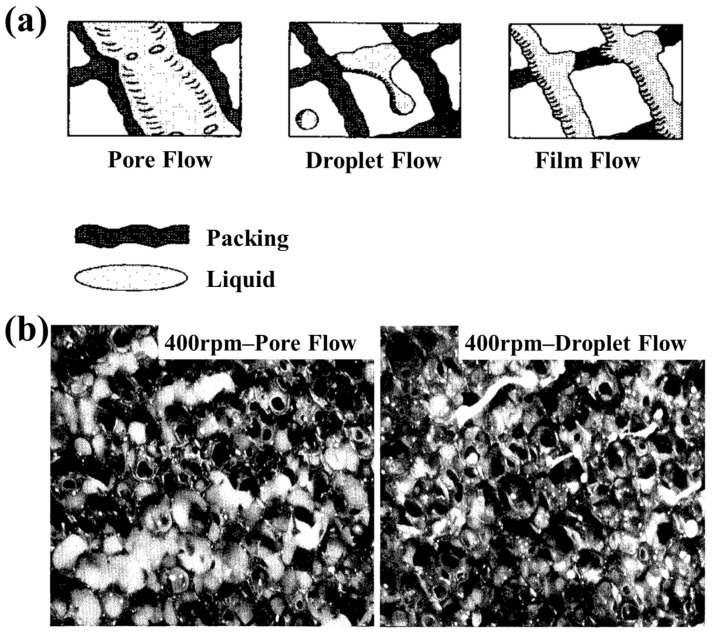
Fluid morphology change in a rotating packed bed [55]. (**a**) The three typical liquid forms; (**b**) morphological differences at different rotational speeds.

When a fluid experiences hypergravity conditions, the *F_c_* (Coriolis force) and interaction force between phases are unavoidable, so fluid dynamics can be modulated, though the properties are unchanged [45,46]. Natural convection relies on the temperature difference or density difference of the fluid to drive the fluid flow from microscale melts and crystal growth [47,48,49,50] to macroscale meteorological convective activities [51,52], while fluid flow and interactions are enhanced under external driving forces. In hypergravity, the convection promoted by hypergravity accelerates mass transfer and relaxes the solute concentration gradient, and natural convection is usually neglected [53,54]. The *F_c_* (Coriolis force), *F_b_* (centrifugal force), and interaction enhance the forced convection, and the direction of forced convection is inconsistent with those of mass transfer and bubble separation. Therefore, researchers have conducted a study on the flow behavior of the fluid in the rotating bed. Burns et al. used high-frequency flash photography to capture the rotating packed bed and found that three liquid flow patterns were dominant: pore flow, liquid flow, and membrane flow, as shown in Figure 3a. Subsequently, it was also found that the form of the liquid was related to the rotational speed of the rotating packed bed: at low rotational speed (400 rpm), the liquid mostly passed through the packing zone in a radial channel flow mode; at high rotational speed (900 rpm), the liquid passed through the packing zone in a droplet state, though the flow form was not discovered, as shown in Figure 3b [55]. 

**Figure 4:**
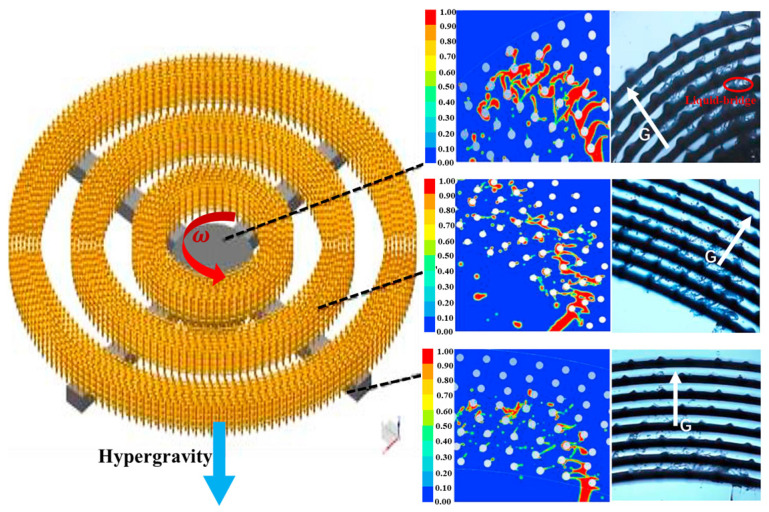
The liquid flow in the MLI-RPB reactor with 3D-printed wire mesh packing was compared with CFD simulation and high-speed camera data (N = 600 rpm) [56].

In a study of the microflow behavior of liquid in a rotating packed bed, Wu et al. [56] 3D printed the segment-inlet filler, combined it with CFD simulation and high-speed camera shooting, and studied the liquid form of liquid flow. As such, they found that in addition to droplets and film, a new type of liquid bridge was observed in the packing ring at lower rotational speeds or in the inner packing ring, which was consistent with the liquid content distribution of different packing rings simulated by CFD. Subsequently, with an increase in rotational speed, the ligament will be shorter, and the probability of the liquid form transforming into the liquid drop in the packing ring will be greater, which indirectly indicates that the higher the level of hypergravity, the more easily the liquid will transform into liquid drop form, as shown in Figure 4.

**Figure 5:**
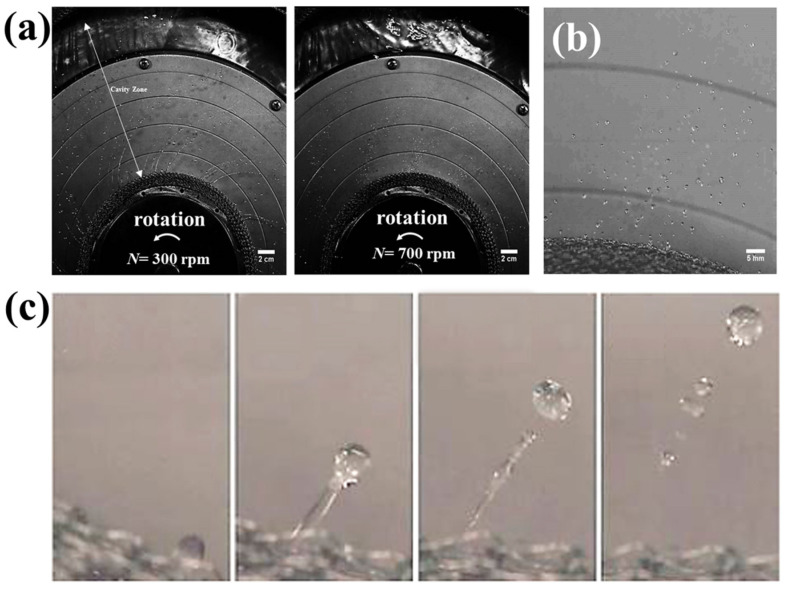
Research on liquid flow behavior in the cavity region of a rotating packed bed [57]. (**a**) Droplet morphological changes in the cavity region at different rotational speeds; (**b**) liquid distribution; (**c**) images of liquid morphological changes. From left to right: The first and the second, the liquid membrane is gradually decomposed into droplets, the third; the droplets are separated from the ligament, and the fourth: the mother droplets are separated to form consecutive droplets.

In addition, the liquid flow pattern in the cavity region of the rotationally packed bed (RPB) is also of great concern. Sang et al. observed two typical liquid flow patterns in the RPB cavity—ligament flow and droplet flow, as shown in Figure 5a—and observed a shift in the liquid flow pattern, i.e., the line to droplet flow transition, as shown in Figure 5b,c. This transition is caused by an increase in rotational speed or a decrease in liquid viscosity, which weakens the effect of viscous damping on the liquid, and by the liquid transitions from the ligament to the droplet [57]. This indirectly suggests that the greater the hypergravity, the greater the number of droplets transformed.

### 3.2. The Liquid-Phase Synthesis of Materials Under Hypergravity 

**Figure 6:**
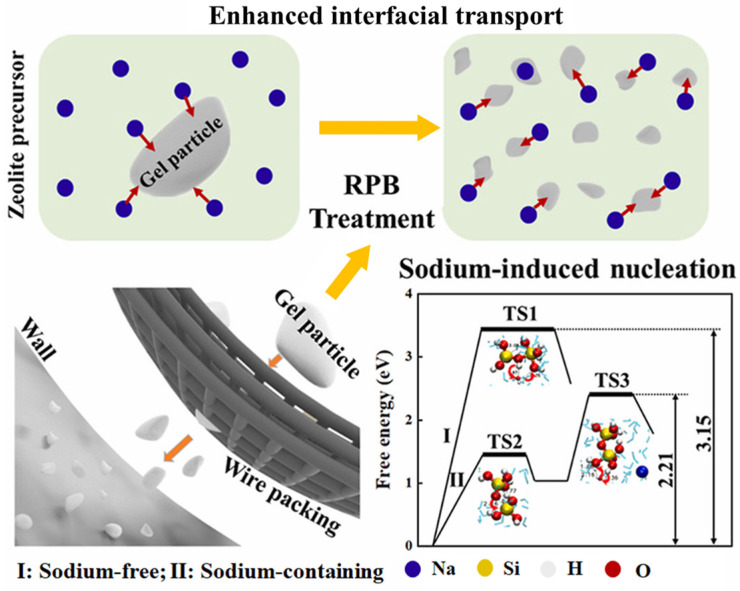
Synthesis of alumina zeolite by rotating packed bed in hypergravity. The red arrow shows the direction of sodium-induced nucleation [63].

Mass transfer and reaction between multiphase fluids are fundamental in industrial processes for liquid-phase material synthesis. The application of hypergravity technology can improve gas–liquid mass transfer in multiphase fluid systems by significantly increasing the rate of interphase transfer and the degree of microscopic mixing between liquid phases [55]. Packing in a hypergravity field induces significant interfacial shear that breaks down liquids into liquid films, filaments, and droplets of micron to nano unit size, continuously refreshing the phase interfaces and thus improving the micro-mass transfer and separation processes between the solution and the precipitant [58]. For example, in a rotary packed bed (RPB), the centrifugal force generated by high-speed rotation can enhance the renewal of the gas–liquid or liquid–liquid interface, thereby improving the efficiency of processes such as absorption, desulfurization, distillation, etc. [59]. Various organic and inorganic nanomaterials, such as SrCO_3_ [60], Mg(OH)_2_ [61], and ZnS [62], etc., have been successfully industrially synthesized. Qi et al. [63] used a hypergravity reactor to develop a new means of rapid synthesis of SiO_2_-alumina zeolites. The presence of a hypergravity force field significantly increased the gel–liquid interface area in the precursor, alleviated the diffusion resistance of zeolites in the initial stage of nucleation, and thus greatly increased the nucleation rate of zeolites and accelerated their growth. In addition, the study also found that hypergravity promoted the transition of hydrated sodium ions and OH^−^ in the liquid phase to the synthetic gel phase, further improving the nucleation efficiency of zeolites. As shown in Figure 6, DFT calculation shows that sodium hydrate can induce Si-OH dehydrogenation to form oxygen free radicals, thus reducing the energy barrier of the synthesis reaction and accelerating the nucleation process.

**Figure 7:**
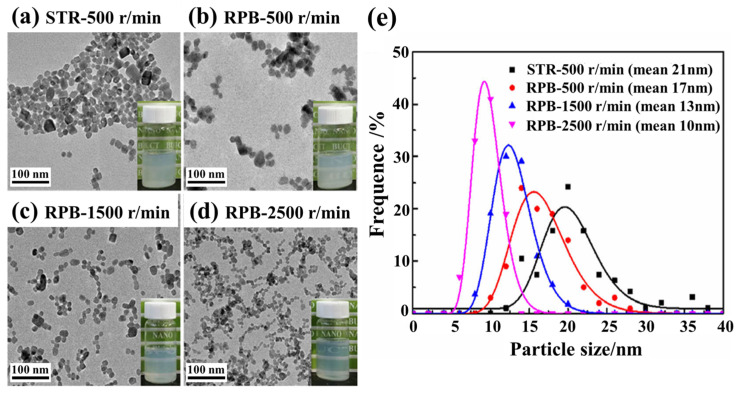
TEM image and particle size distribution of BaSO_4_ nanoparticles prepared at different rotational speeds. (**a**) The stirred tank reactor uses a rotational speed of 500 r/min (**b**) RPB uses a rotational speed of 500 r/min (**c**) RPBuses a rotational speed of 1500 r/min (**d**) RPB uses a rotational speed of 2500 r/min (**e**) Particle size distribution [64].

Similarly, Figure 7 shows the results of a study on the preparation of barium sulfate nanodispersions by precipitation in a RPB, which found that the overall particle size was about 10 to 17 nm according to the transmission electron microscope (TEM) analysis, and the product prepared using RPB had a smaller particle size and a narrower particle size distribution than the barium sulfate prepared in a traditional stirred tank reactor (STR) [64].

### 3.3. The Separation of Materials Under Hypergravity 

Starting with the commonalities among the separation and purification of materials, the basic theories of the equilibrium separation and distillation of substances in a hypergravity environment have been established [65,66]. Combined with the process intensification and coupled separation behaviors, a complete set of process technologies, such as gas purification, gas–solid separation, liquid–liquid separation, and controlling and separating pollutants in water, can be developed using hypergravity technology [67]. In the process of smelting slag at a high temperature, the metal droplets brought about by the violent reaction will be mixed with the slag, thus discharging the slag and causing waste. Valuable metals in the slag in the form of droplets gather with a difficult and diffuse distribution, which is mainly due to the droplets having high surface tension and the buoyant force being too small.

Under hypergravity conditions, the distinct buoyancy forces result in differing distribution trends for the two phases, thereby achieving phase separation, which is known as centrifugal separation. The increased acceleration and enhanced convective effects accelerate the migration speed between phases of different densities, leading to an enhanced separation rate. In contrast, under normal-gravity conditions, phase separation is driven by diffusion and buoyancy effects, with a relatively slower speed. Therefore, hypergravity technology can effectively realize the separation of the liquid phase.

**Figure 8:**
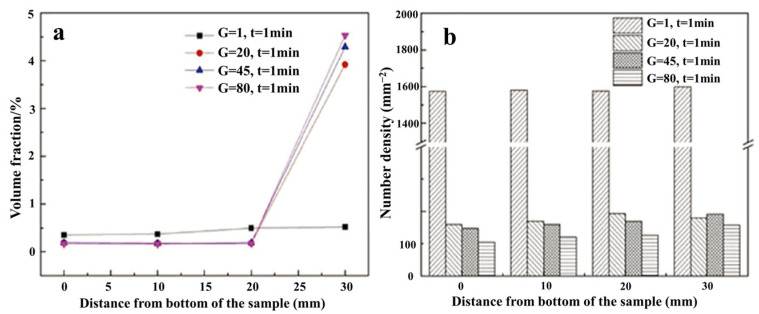
The growth of SiO_2_ inclusions in 304 stainless steel after treatment for 1 min under different gravity conditions [70]. (**a**) Volume fraction; (**b**) density distribution.

In the preparation process of high-end metal materials, the presence of non-metallic inclusions will destroy the uniform distribution of the substrate, thus affecting the comprehensive properties of the material. Traditional refining processes, such as electromagnetic stirring, gas stirring, etc. [68,69], have limited purification capacity in liquid metal, and it is difficult to completely remove inclusions. Ideally, as the gravity coefficient increases, the inclusions will rise to the top of the sample, as shown in Figure 8, where the SiO_2_ inclusions in 304 stainless steel are evenly distributed under normal gravity, while the volume fraction and quantity density of SiO_2_ are distributed in gradient after the introduction of hypergravity, causing them to migrate to the top of the sample along the reverse direction of hypergravity [70]. 

**Figure 9:**
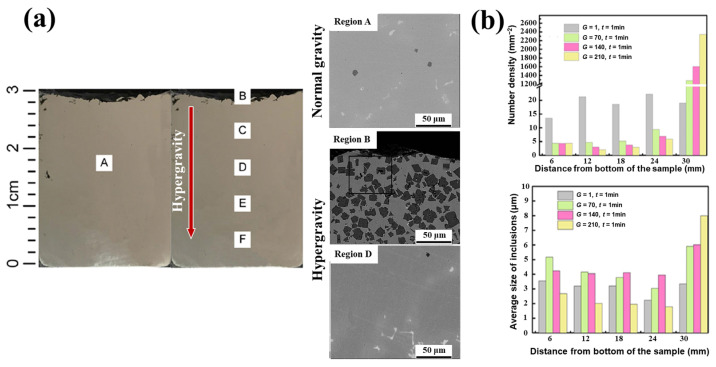
Hypergravity-enhanced separation for the removal of inclusions from Inconel 718 superalloys [73]. (**a**) Macroscopic and microscopic imaging of samples before and after treatment under hypergravity; (**b**) density and particle size distribution of inclusions after treatment of Inconel 718 superalloys under different hypergravity conditions (A: The central area of the ingot under normal gravity; B–F: Different areas of sample cross section obtained by hypergravity facilitation separation).

Similarly, studies on the removal of inclusions by hypergravity in aluminum alloys and copper alloys show that the removal of inclusions by hypergravity is very effective [71,72]. As shown in Figure 9, the inclusions in molten-state Inconel 718 superalloys were concentrated on the top of the sample under the treatment of different gravity conditions, and there was almost no bottom of the sample. The size distribution of the inclusions under normal-gravity conditions (G = 1) was relatively uniform, concentrated in 2~4 μm, while in the hypergravity condition (G = 70~210), the average size of the inclusions on the top of the sample was 7~9 μm, which was much larger than that of the middle and lower part of the sample [73]. It can be seen that the main task in melting recycling alloy materials is to remove inclusions, and the separation processes of hypergravity settling/uplifting or filtration are efficient means of doing so.

**Figure 10:**
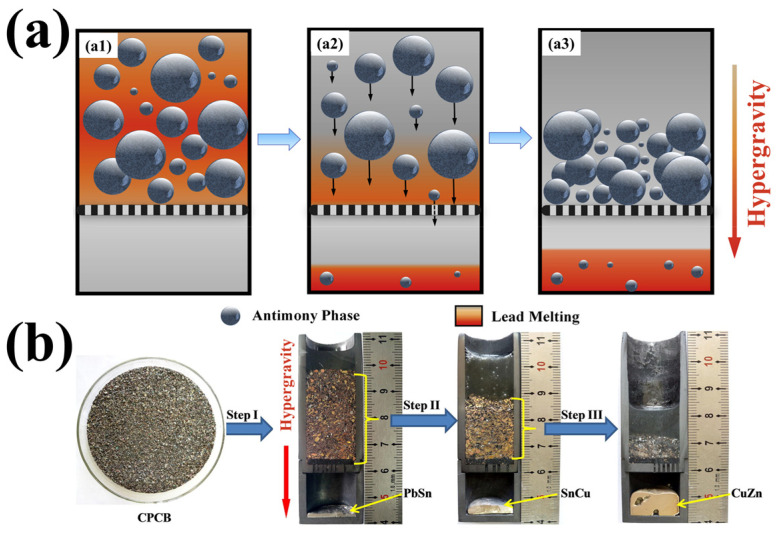
Hypergravity separation of valuable components in mixed metals [39,74]. (**a**) Schematic diagram of filtration mechanism of hypergravity technology (**a1**): The initial phase of random distribution of antimony phase,(**a2**): Hypergravity is applied for separation, (**a3**): The phase with small particle size is separated to the bottom, black arrow: direction of phase migration); (**b**) Hypergravity technology used for fractional separation and recovery of Pb, Sn, Zn, and Cu.

Hypergravity technology plays an important role in the enrichment and separation of valuable elements. In the selective separation, enrichment, and recovery of valuable metals in non-ferrous metal ores, it is important to clarify the reaction and separation behavior of multi-metal components to achieve efficient separation. By utilizing the heterogeneous mass transfer and strong phase migration of the hypergravity field, electronic waste is placed in a graphite crucible and a filter, as shown in Figure 10a, for heating and applying hypergravity. The phases with different melting points will be sequentially separated to the bottom, as shown in Figure 10b [39,74].

For example, in waste printed circuit boards (WPCBs), there are valuable metals such as gold and silver, and failing to recover them will lead to serious soil pollution problems. Traditional methods can utilize the difference in vapor pressure of various metals to purify the processed granular metal by combining evaporation and condensation technology (such as Pb and Zn) [75]. Low-temperature alkaline smelting technology can also be used to extract valuable metals from mixed-state metals, which has a good purification effect on amphoteric metals such as Sn, Pb, Al, and Zn [76]. However, these two methods are not suitable for the purification of other metals in WPCBs. When a mixed-metal circuit board is heated to a molten state under normal gravity, the surface tension between different metals hinders their separation. Under hypergravity conditions, however, various metals can be separated according to their different melting points.

**Figure 11:**
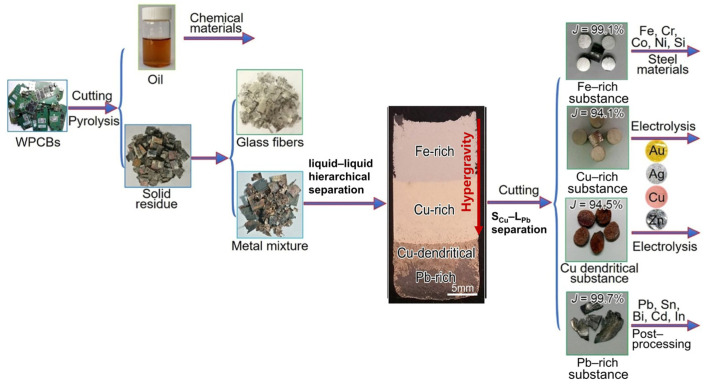
WPCB metal recovery process combining the pyrolysis process and hypergravity technology separation [75].

Chen et al. [75] used the pyrolysis method to manually dissociate the mixed metal and glass debris of WPCBs and then heated the mixed metal to 1400 °C under 1000× *g* hypergravity conditions. The ingot along the hypergravity direction had Fe-rich, Cu-rich, Cu-rich, and Pb-rich layers, and the separation rate between the mixed metals could reach more than 94%, as shown in Figure 11, This suggests that hypergravity is conducive to the separation of different metals.

The advantages of hypergravity in accelerating the separation of substances with different densities have been extensively utilized in isotope separation, significantly contributing to the advancement of science and technology [77]. For instance, the gas-phase centrifuge separation of nickel isotopes ^62^Ni and ^64^Ni, which form gaseous molecules at near-ambient temperatures, has expedited the development of medical technology [78]. However, isotopes that cannot be vaporized at ambient temperatures, such as group I (alkali metals) and II (alkaline earth metals) elements, are not suitable for gas-phase separation techniques. Notably, the liquid centrifugal method can be employed to separate and extract isotopes of almost all the elements. This method operates under conditions close to ambient temperature and pressure, avoiding the use of harmful gases. The heavier isotopes concentrate at the outer edge of the centrifuge, while the lighter isotopes accumulate at the inner edge [79]; such a phenomenon is also observed in the solid phase [80]. Nevertheless, there are challenges associated with this process; centrifugal separation equipment must withstand the immense centrifugal force generated by high-speed rotation, requiring materials with exceptional strength and corrosion resistance.

## 4. Metallurgy of Alloys Under Hypergravity 

### 4.1. Convection and Segregation Under Hypergravity 

The solidification of the alloy melt causes differences in the composition of the solute, meaning the concentration gradient will cause the natural convection of the melt. Solidification and melt flow are strongly coupled, and this is reflected in the solute removal during the crystallization process of the primary crystal, which causes solute pair flow at the solid–liquid interface, and the melt convection in turn affects the growth rate and direction of the crystal [81,82]. In addition, the morphology of the grains during crystal growth will be affected by the melt flow, which is mainly caused by the solute pair flow [83,84]. The influence of alloy melt convection on the solidification process under a normal-gravity field has been extensively studied, as well as in microgravity or hypergravity field experiments [85,86,87,88,89]. The alloy melt will show different flow states under the action of different gravity fields. The convective instability of the liquid phase can be measured according to a physical quantity, namely Rayleigh number *Ra*, which is expressed as outlined in Equation (7) [5]:

(7) $$Ra=\frac{\beta g∆L^{3}}{\nu k}$$

where *g* is the acceleration of gravity, *β* is the isobaric thermal expansion coefficient, ν is the kinematic viscosity, *κ* is the thermal diffusivity, Δ is the temperature gradient between the hot and cold plates, and *L* is the thickness of the fluid layer between the above plates. The melt does not flow when the Rayleigh number is small. When the Rayleigh number exceeds a critical value, the buoyancy overcomes the viscous force, and natural convection occurs. Under the conditions of natural convection, when the Raleigh number is small, it is a laminar flow. With an increase in Rayleigh number, the change from laminar flow to turbulent flow will occur [90]. In the hypergravity field, the Rayleigh number further increases, and the convection state returns from turbulence to laminar flow, also known as “re-laminarization”. However, under the conditions of centrifugal force and buoyancy introduced in the hypergravity force field, the Coriolis force generated by rotation around the centrifugal axis will change the flow state of the melt. In particular, when the Coriolis force is coupled with a stable axial temperature gradient, it mainly redistributes the convection in the melt [91,92], while in the case of an unstable temperature gradient, the Coriolis force promotes the basic rearrangement of the flow [93]. At present, scientists use the Rossby dimensionless number Ro to evaluate the influence of the Coriolis force on convection and describe the influence of the Coriolis force in the centrifugal rotating system. Under a hypergravity field, convection is strengthened, and Rossby, *Ro*, is expressed as outlined in Equation (8) [94]:

(8) $$R_{O}=\frac{U}{\Omega L}=\frac{\nu G_{r}^{\frac{1}{2}}}{\Omega L^{2}},$$

where *ν* is the kinematic viscosity of the liquid, *U* is the characteristic velocity, Ω is the rotational angular velocity, *L* is the characteristic length, and *Gr* is the Grashof number. In general, the Rossby number is greater than 1, indicating that the influence of the Coriolis force can be ignored.

When the Rossby number is much less than 1, the Coriolis force completely determines the flow pattern of the fluid. Cisternas Fernández et al. [95] showed the deflection effect of the Coriolis force on alloy melts in experiments where all Rayleigh numbers were much lower than 1. In this case, the Coriolis force pushes the lighter liquid toward the sample’s flight speed, resulting in a large circular backflow throughout the entire liquid region. However, in Battaglioli’s dimensionless analysis [96], Coriolis forces increase proportionally to the ratio of buoyancy brought about by hypergravity sites, and their magnitude is negligible relative to the buoyancy caused by centrifugal acceleration.

The metallurgical process that materials undergo is a complex liquid–solid phase transition reaction, accompanied by a series of physical and chemical reactions such as heat loss, melt convection, and solute redistribution [97]. The solidification interface is affected by heat or solute redistribution, resulting in dendritic growth during solidification [98]. The distribution of the solute may lead to the formation of harmful casting defects such as “freckles” in the microstructure of metal alloys [99]. These “freckles” have been observed in many materials, such as directionally solidified turbine blades, cast ingots, etc. The convection driven by the buoyancy of the hot melt of an alloy liquid will lead to the uneven distribution of solute between solid and liquid phases in a specific thermodynamic state, which is one of the reasons for the segregation. The segregation behavior of the solidification process is often accompanied by a gradual change in composition. Numerous studies have shown that segregation and melt convection have a direct effect on the formation of casting defects [100,101,102]. 

When the gravitational acceleration is much less than the Earth’s gravitational acceleration (g ≈ 9.81 m/s^2^), the environmental state is called a microgravity field. At this point, the gravitational effect on the object is negligible, putting the object in a near-weightless state. The buoyancy effect of gravity causes solid particles to settle or float during solidification [103]. The magnitude of gravity makes it difficult to understand the equiaxed crystal transformation that occurs during solidification, while natural convection and sedimentation effects are greatly weakened or disappear in microgravity [104,105]. During solidification, solute diffusion and crystal growth are mainly controlled by the diffusion process rather than convection [106,107]. This is conducive to improving the nucleation and growth conditions of equiaxed crystals. For example, in the dendrite growth of Ni-50%Cu supercooled melt under microgravity and with no container, the crystal growth morphology changes from coarse dendrites to uniform equiaxed crystals with an increase in the supercooling degree [108].

The buoyancy drive of the centrifugal casting process will be affected by the acceleration of the non-inertial frame, which is due to the combined effect of centrifugal acceleration and the Coriolis force. In the hypergravity field, there is a certain centrifugal force gradient in the alloy melt in different regions of the rotating packed bed, which may aggravate the flow behavior, make the fluid state transition from laminar flow to turbulent flow mode, and finally cause it to reverse back to laminar flow mode [109]. When centrifugal casting turbine blades of high-Nb TiAl alloy [110], it was found that dendrite γ segregation occurred at the edge of the casting surface due to rapid cooling. This is because hypergravity increases the equivalent interfacial heat transfer coefficient between the alloy melt and the die, increasing the cooling rate of the surface [111].

**Figure 12:**
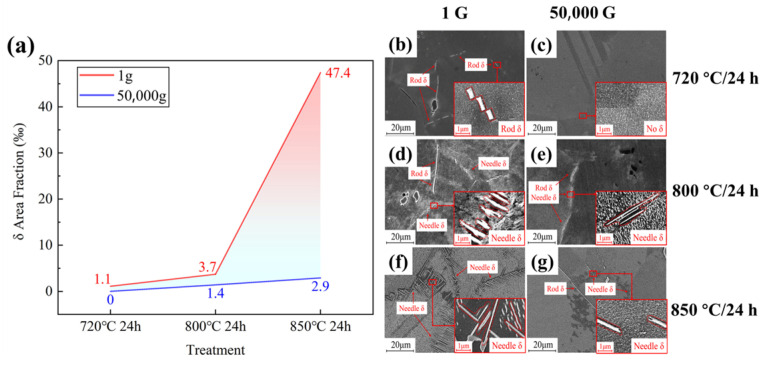
Distribution and SEM of the precipitated phase in GH4169 alloy under diverse gravity conditions and temperature treatments [112]. (**a**) Statistics of the δ phase area fraction; (**b**) SEM images of the rod-shaped δ at 720 °C, 24 h, 1 G; (**c**) SEM images showing no δ at 720 °C, 24 h, 50,000 G; (**d**) SEM images of the needle-like δ at 800 °C, 24 h, 1 G; (**e**) SEM images of the needle-like δ at 800 °C, 24 h, 50,000 G; (**f**) SEM images of the needle-like δ at 850 °C, 24 h, 1 G; (**g**) SEM images of the needle-like δ at 850 °C, 24 h, 50,000 G.

High temperatures affect the diffusion rates, chemical reaction rates, and thermodynamics within materials, while hypergravity influences interphase interaction forces and stresses. Therefore, for certain material systems, there may exist specific temperature ranges where these effects are most pronounced. Under a hypergravity force field, convection and segregation will result in the inhibition of nucleation and precipitation of the alloy. As shown in Figure 12, a microstructural analysis conducted using scanning electron microscopy (SEM) reveals the morphology and variations in area fraction of the precipitated phase δ at the grain boundary of GH4169 superalloy after a centrifugation experiment. It was discovered that [112] under normal gravity of 1× *g*, as the temperature increases (720–800–850 °C), the amount of δ phase precipitation of GH4169 alloy gradually rises, and the morphology changes from rods to large-angle needles. At 50,000× *g*, the distribution and area fraction of the δ phase decrease. The precipitation of the δ phase is inhibited by the hypergravity level, and the inhibitory effect intensifies with the increase in aging temperature. The mechanism of inhibition is that hypergravity reduces the possibility of lattice distortion and internal accumulation fault during the precipitation of the γ phase from the matrix, thus reducing the nucleation site of the δ phase.

### 4.2. Grain Refinement of Solidification 

**Figure 13:**
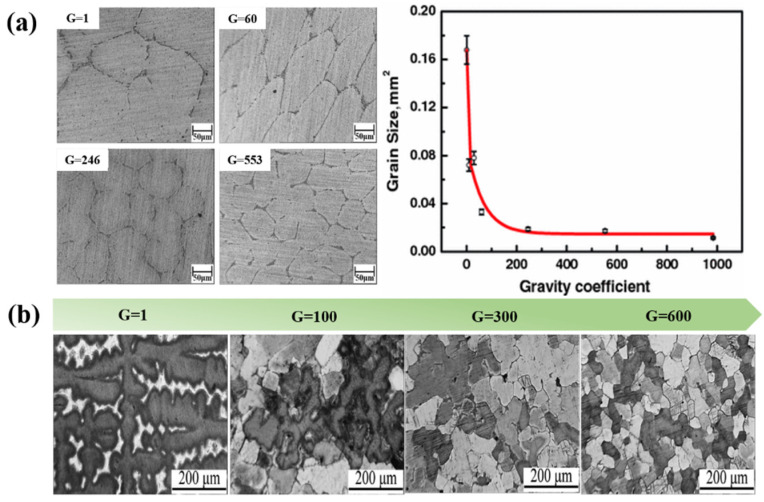
Effect of hypergravity coefficient on solidified grains of different metals [113,114]. (**a**) Pure aluminum; (**b**) Cu-11%Sn alloy.

As we all know, the grain size of metal materials significantly affects their mechanical and processing properties: the finer the grain, the higher the strength of the material. The grain refinement process of cast parts has been widely reported in previous studies. Hypergravity has a non-negligible role in the solidification process. As shown in Figure 13a, the influence of the hypergravity coefficient on the grain size during the solidification process of pure aluminum, under the same temperature conditions, shows that the larger the hypergravity coefficient, the more significant the effect of grain refinement [113]. Yang [114] studied the solidification process of Cu-Sn alloy under hypergravity conditions. The results show that the higher the gravity coefficient, the more significant the refinement effect on the solidification structure. Hypergravity causes the solidification structure to change from coarse columnar crystals and developed dendrites to fine, equiaxed crystals and spherical crystals, as shown in Figure 13b. It is proposed that hypergravity affects grain refinement not during the melt or grain growth stages but during the nucleation stage. Zimmermann et al. [115] used transparent model alloy C_5_H_12_O_2_-C_10_H_16_O to observe the increase in nucleation rate and the precipitation of dendritic crystals during solidification under a hypergravity field.

**Figure 14:**
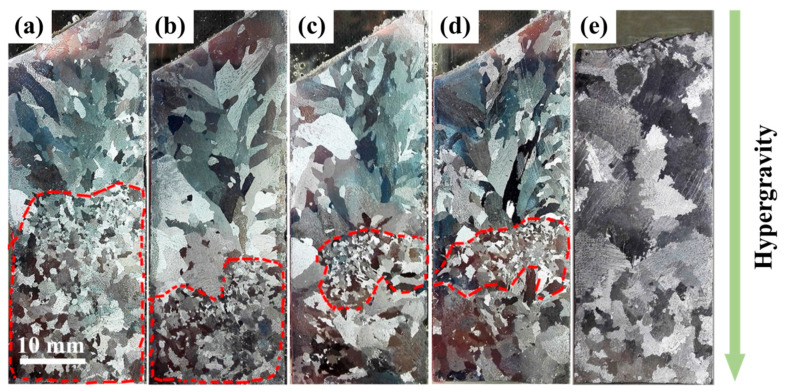
Microstructure of Al-8 wt% Cu alloy after hypergravity treatment with G = 300 at different solidification stages [116]. (**a**) Starting from high-temperature liquid and ending with a solid phase fraction of 20%; (**b**) Starting with a solid phase fraction of 20% and ending with a solid phase fraction of 50%; (**c**) Starting with a solid phase fraction of 50% and ending with a solid phase fraction of 80%; (**d**) Starting with a solid phase fraction of 80% and ending with a solid phase fraction of 100%; (**e**) The complete solidification stage, where the red wireframe represents the fine crystal zone (Red dotted line: the fine grain zone of each sample).

During metal solidification under normal-gravity conditions, the gravity difference between the crystalline droplets and the molten liquid phase is minimal, resulting in very slow movement between the two phases. The presence of the hypergravity field makes the high-density metal particles overcome the resistance caused by the liquid phase. A large gravity difference between the two phases causes the crystalline particles to continue moving in the direction of hypergravity, resulting in the phenomenon of “crystal rain” [116] in the molten metal, which promotes the proliferation of crystal nuclei and further refines the grains. As shown in Figure 14, hypergravity-field-induced grain refinement occurs in the early stages of solidification, with the fine-crystal region gradually moving in the opposite direction to hypergravity in the later stages of solidification. 

**Figure 15:**
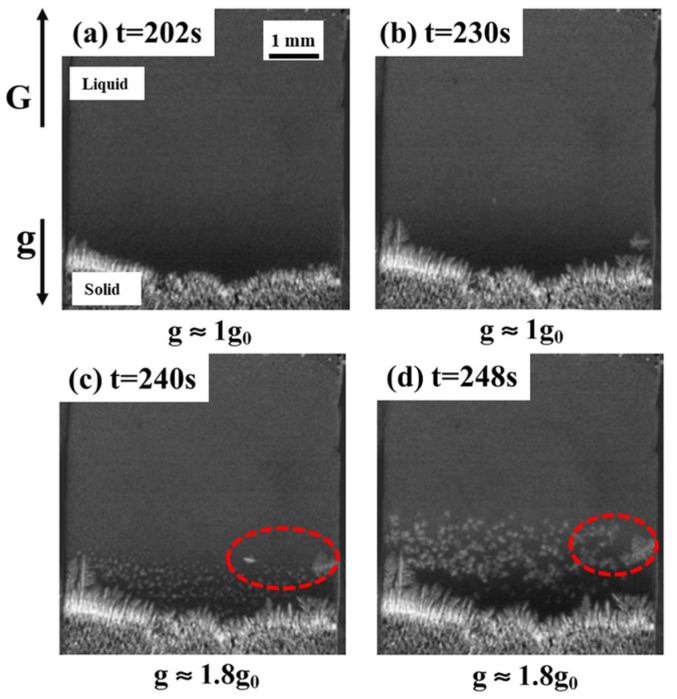
Evolution of dendrite structure at solid–liquid interface under different gravity conditions. Red dashed circle: broken dendrites [121].

In addition, dendrites generated during the casting process form new nucleated nuclei, which are reinforced by the intervention of external fields, such as electromagnetic fields and ultrasonic waves [117,118]. The strong convection phenomenon caused by the hypergravity field will also locally alter the subcooling degree of the melt (dendrites), resulting in changes to the nucleation mode and growth rate of the liquid phase during crystallization [119]. Phase-field simulations of mono-crystalline and polycrystalline materials also demonstrate that hypergravity can promote dendrite growth direction and fragmentation [120]. As shown in Figure 15, an X-ray system was used to observe the changes in the solidification process of Al-20 wt% Cu. It was found that the dendrites broke along the solid–liquid interface, and the fragments floated from the bottom to the top due to buoyancy. The dendrite fragments melted rapidly in the hot region of the specimen until they disappeared completely. When hypergravity reaches 1.8 g, more equiaxed grain nucleation appears at the front of the columnar crystal [121]. The increase in heterogeneous nucleation sites caused by dendrite fragmentation plays a key role in grain refinement. 

**Figure 16:**
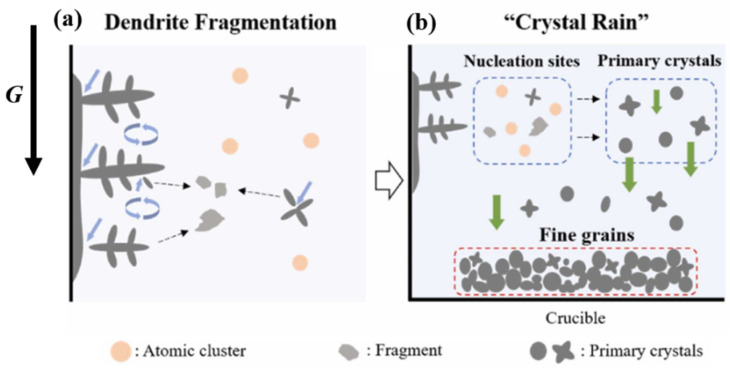
Mechanism of grain refinement of 7050 aluminum alloy under hypergravity (**a**) Dendrite fragmentation. (**b**) Dense primary crystals settle to the bottom (blue arrow: hypergravity causes the dendrite root to break; dashed arrow: broken dendrites begin to form primary crystals; green arrow: direction of settling of primary crystal) [122].

You et al. [122] found that in centrifugally cast Al7050 aluminum alloy, the hypergravity force field enhanced the weak parts of dendrites (Figure 16a, blue arrow), resulting in dendrite fragments of different sizes, which were used as the sites of heterogeneous nucleation, thus improving nucleation efficiency. Hypergravity causes the initial crystal and dendrite debris to settle at the bottom (Figure 16b, green arrow). The broken dendrites are recognized as the new nucleation sites in the mechanism of grain refinement under the strong convection conditions brought about by the hypergravity.

However, the specific effects of a hypergravity field on dendrite morphology and size during nucleation, including but not limited to the initial formation of dendrite, growth direction, branching characteristics, etc., still need to be further studied.

### 4.3. The Dendrite Structure of Alloy Under Hypergravity Conditions 

**Figure 17:**
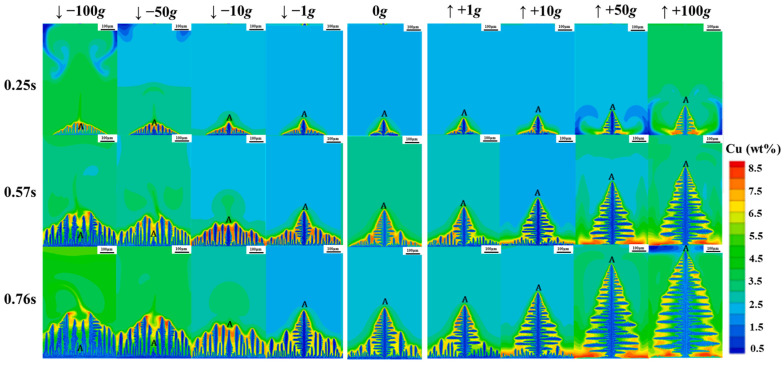
The growth process of columnar crystals under different gravity conditions. A indicates the primary dendrites; the direction of gravity vector is the same as that of main dendrite growth under positive gravity and the opposite direction under negative gravity; blue is the primary dendritic phase, and green or red is the copper-rich liquid; the scale is 100 μm [126].

In the actual process of crystal growth, the intervention of an external field can control the growth form of the crystal, its evolution can lead to the formation of a cellular interface due to the instability of the crystal surface, and further instability will cause dendrite growth. Moreover, dendrite growth in alloy materials can produce micro-segregation and other internal defects in castings and ingots, thus affecting the performance of castings [123]. For example, the primary dendrite arm spacing affects the hardness of the casting [124]. The hypergravity field simulated in the centrifugal casting process exacerbates the buoyancy-driven flow behavior and thus alters the alloy melt mass and heat transfer processes. When the solidification of molten metal in a hypergravity field occurs at certain nucleation points, the growth of dendrites and the morphology of the solidified structure are related to heat and mass transfer. The pressure on the liquid in a hypergravity field is anisotropic, which may cause the anisotropy of heat and mass transfer, which in turn affects the growth of dendrites and the morphology of solidified tissues; e.g., when the direction of hypergravity is opposite to the upward-growing dendritic dendrites, the primary dendritic arm spacing (PDAS) decreases with an increase in the hypergravity level [125]. As shown in Figure 17, phase-field simulation in Al-Cu alloys reveals that the dendrite growth morphology is related to the magnitude and direction of gravity. Under positive gravity, the primary dendrites grow longer with an increase in hypergravity, the growth of other secondary dendrites becomes shorter and shorter, and the growth of bottom dendrites is completely inhibited at +50 g. Under negative gravity, the increase in hypergravity makes the length of primary dendrites shorter and shorter, while the number of branches of secondary dendrites and other dendrites increases [126]. The change in the direction of positive gravity is related to the melt flow pattern caused by hypergravity. In the enhanced convection mode, the flow caused by the density difference is downward, and the latent heat of the melt is released to the bottom, making the concentration and temperature gradients larger, and thus promoting the growth of the dendrite tip [127]. It is worth noting that in the centrifugal casting of Ti-Al alloys, the effect of hypergravity on the initial dendrites obtained from numerical simulations is different [128]. Therefore, the processes related to dendrite growth during solidification under a hypergravity field need more reliable experiments to validate the simulation results.

**Figure 18:**
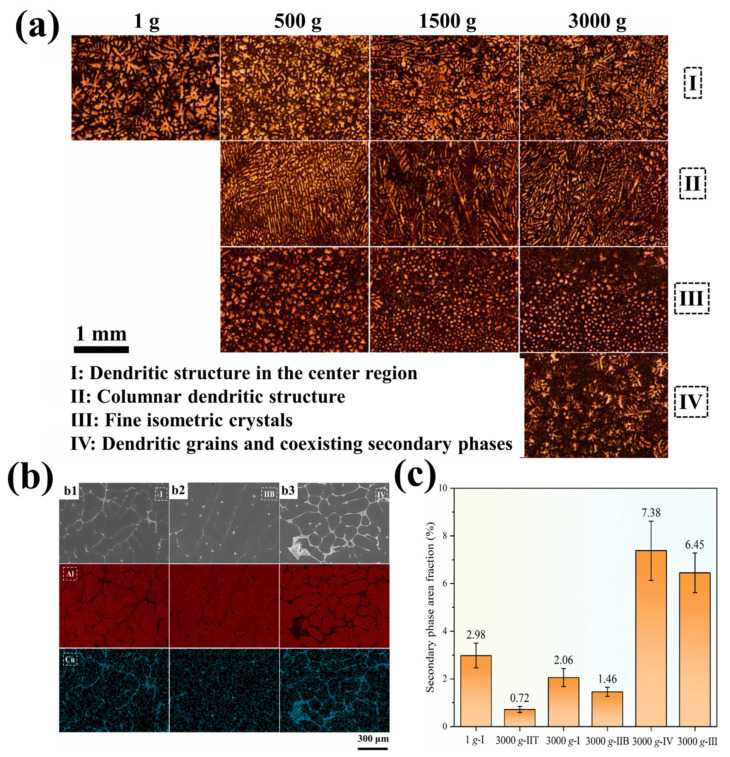
Effect of hypergravity on dendrite growth [122]. (**a**) Dendrite structure at different locations; (**b**) the typical element distribution and microstructure of the second phase of Al7050 alloy at 1023 K (**b1**): 1 g—region I, (**b2**): 3000 g—region II, (**b3**): 3000 g—region IV. I–IV represent different regions). (**c**) area fractions of the second phases of different structures, B and T represent the bottom and top regions, respectively.

In the experiment, You et al. [122] studied the centrifugal casting process of Al7050 aluminum alloy and found that obvious microstructure changes occurred along the direction of hypergravity, resulting in different types of dendritic regions (I-II-III-IV regions, as shown in Figure 18a; IIT and IIB are, respectively, the top and bottom II structures). It was found that type-II columnar dendrites appear under hypergravity and grow gradually with an increase in the hypergravity coefficient. As shown in Figure 18b,c, from the area and elemental distribution of the second phase between dendrites, it can be observed that the strong convection caused by hypergravity will push elements, such that the concentration gradient at the tip of the dendrites becomes too large, accelerating the dendrite growth process. Once the solute concentration reaches dynamic equilibrium, it inhibits the further growth of the columnar crystals, resulting in a type-IV structure with a higher secondary phase area fraction.

### 4.4. The Gradient Structure Under Hypergravity Conditions 

**Figure 19:**
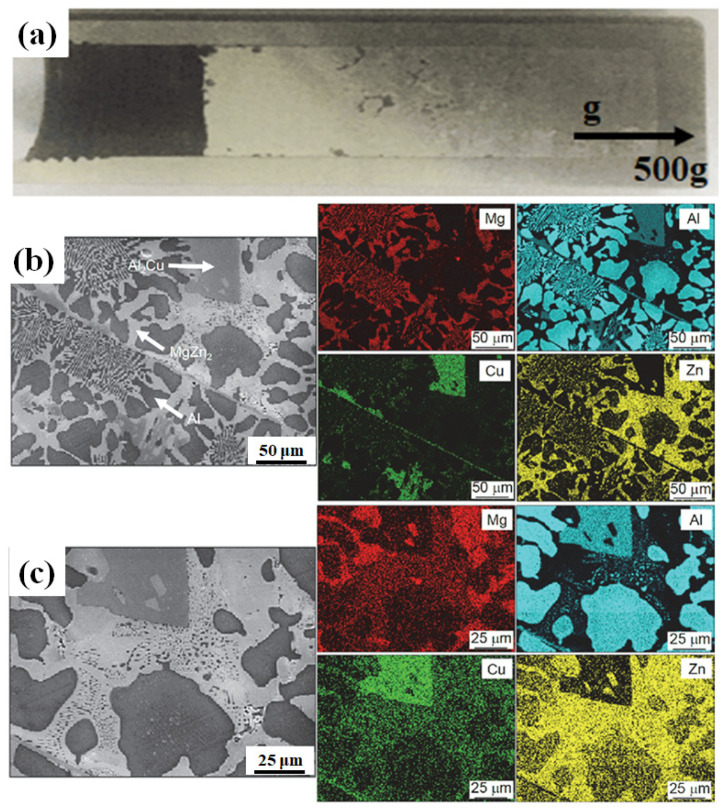
The gradient structure was prepared under hypergravity [134]. (**a**) Samples after hypergravity treatment; (**b**) low-magnification and (**c**) high-magnification SEM and EDS analysis for the AlZn_0.4_Li_0.2_Mg_0.2_Cu_0.2_.

For fast sample preparation and rapid optimization of performance, conventional metal materials can be specially prepared as gradient functional materials with gradient changes in composition or properties. Gradient materials with continuous variations in composition, structure, and properties in specific directions require one of the methods of high-throughput preparation [129,130]. Laser fusion deposition [131], self-propagating high-temperature synthesis [74], chemical vapor deposition [132], and many other methods have been successful in preparing gradient functional materials, and hypergravity technology has been equally effective [133]. As shown in Figure 19, in a hypergravity field of 500 *g*, the microstructure of the alloy undergoes a large change in phase morphology along the direction of hypergravity [134], and the apparent gradient structure is due to the effect of the gradient distribution of trace alloy oxides on nucleation during centrifugation. As the gravity field reaches 60,000× *g*, an obvious crystalline phase delamination phenomenon occurs in the Mg_56_Al_30_Li_7_Cu_7_ metallic glass organization, and the high-gravity region is replaced by a new copper-rich binary eutectic organization, which reveals the solidification sequence in multicomponent alloys under a hypergravity field and provides a guideline for the development of new bulk metallic glass materials [135].

## 5. Structural and Properties of Alloy Materials Under Hypergravity

### 5.1. Solid-State Diffusion Under Hypergravity Conditions

Elemental diffusion is a phenomenon of atomic migration in solid materials. This process is based on Gibbs free energy or chemical potential [136]. Under normal-gravity conditions, the most common diffusion mechanism in solid-phase alloy systems is vacancy diffusion, i.e., when atoms migrate and diffuse through vacancies in the crystal lattice, a mechanism that has been confirmed by numerous experiments, including the famous Kirkendall void phenomenon [137]. In a normal-gravity field, the diffusion coefficient is related to the product of the vacancy concentration and its jump probability [138]. Under the applied stress field, diffusion parameters such as the lattice constant, hopping mechanism, and vacancy activation energy will change, thus changing the diffusion behavior [139,140]. When applying conventional stress conditions under normal gravity, Cowern [141] and Kringhøj [142] proposed a stress-based pair. The vacancy diffusion mechanism model outlines the influence of atomic diffusion activation energy. The basic concept of this model is that when the material is subjected to tensile stress, the activation energy for atomic diffusion increases, and the atomic diffusion is inhibited. In the case of compressive stress, the change in activation energy is opposite due to the decrease in diffusion activation enthalpy, which promotes atomic diffusion.

The hypergravity environment differs significantly from the conventional stress environment experienced under normal-gravity conditions. Early research involved fundamental studies on the effects of high-temperature hypergravity environments on solid-phase materials, conducted by simulating hypergravity using devices such as centrifuges with integrated heating. These studies revealed that hypergravity fields influence the diffusion of elements within solid-phase alloys. For instance, investigations of gold–potassium (Au-K) alloys at different hypergravity levels (achieved by changing the speed of the centrifuge) have shown that gold atoms with larger atomic masses exhibit faster diffusion rates [143].

**Figure 20:**
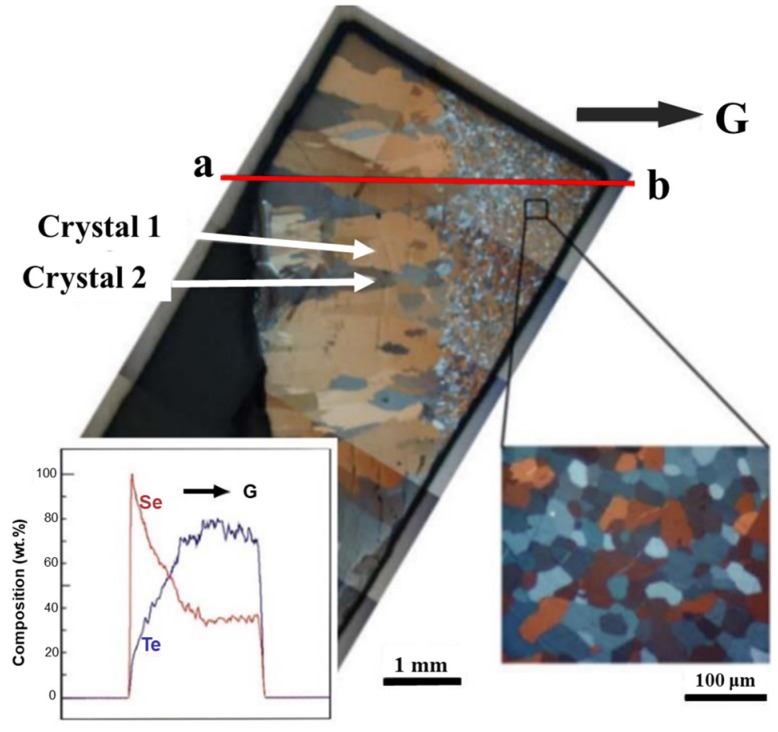
Gradient structure in Se-Te alloy after hypergravity treatment. a–b solid red line indicates the direction of component detection [144].

This phenomenon is attributed to the centrifugal force induced by hypergravity, which increases the chemical potential of the solution composition and promotes the deposition of heavier gold atoms. Atomic deposition with a higher atomic mass has also been confirmed in selenium–tellurium (Se-Te) alloys [144], such as gradient structures with different lattice constants formed along the direction of hypergravity, as shown in Figure 20. Therefore, hypergravity enhances the atomic diffusion of heavier species.

**Figure 21:**
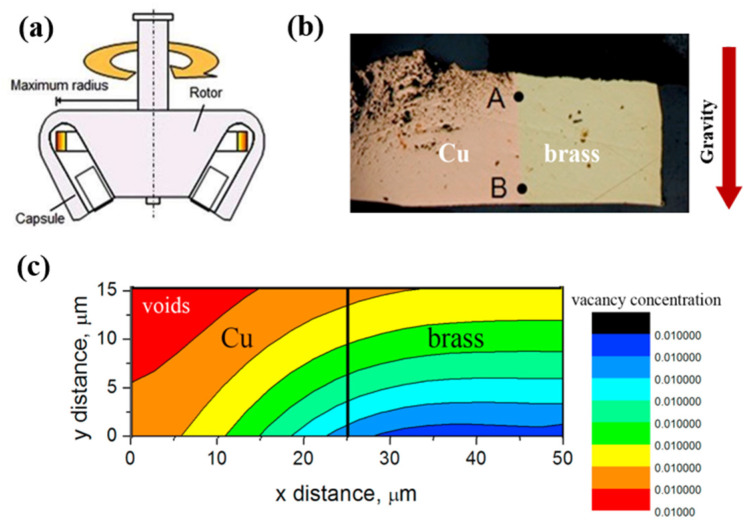
Effect of high-temperature hypergravity on copper–brass diffusion. (**a**) Centrifuge-modified high-temperature hypergravity environment equipment [145]; (**b**) copper–brass diffusion couple treated with high-temperature hypergravity (400 °C, 400,000× *g*); (**c**) approximate vacancy concentration distribution in copper–brass diffusion couple after high-temperature and hypergravity treatment. Points A and B represent the top and bottom of the sample [146].

Studies of the interface between copper (Cu) and brass (CuZn) alloys [145,146] show that hypergravity affects the distribution of vacancy concentrations in the material and thus the diffusion of elements in the alloy, as shown in Figure 21. It can be seen that the diffusion of heavier atoms can be enhanced under a hypergravity field, which does not occur under normal-gravity conditions. It is worth noting that hypergravity is a combination of centrifugal force, buoyancy force, and the Coriolis force, so the influence of hypergravity on diffusion cannot be simplified to traditional tensile stress or compressive stress, and the influence of centrifugal force and buoyancy on the diffusion system of binary alloys must be further studied.

**Figure 22:**
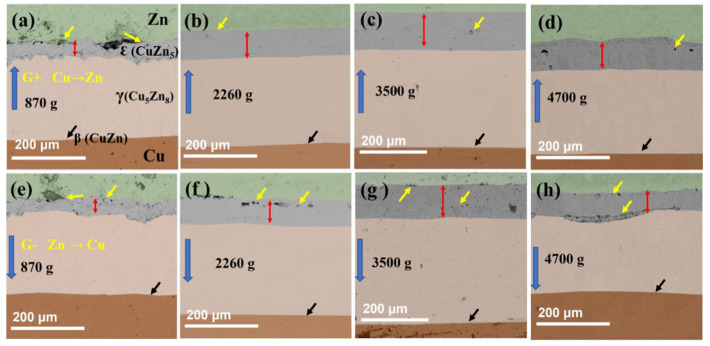
Backscattering SEM images of the sample interface at a hypergravity level of 870, 2260, 3500, and 4700× *g*, with diffusion at 350 °C for 6 h [147]. (**a**–**d**) G+ sample, the hypergravity direction is Cu to Zn; (**e**–**h**) G- sample, the hypergravity direction is Zn to Cu. The yellow arrows indicates the defects (voids), the red arrows indicate the ε phase diffusion layer, and the black arrows indicate the β phase (not obvious).

Some studies have evaluated the reliability of this simplification. By studying CuZn diffusion pairs treated under hypergravity, they found that the mutual diffusion behavior of an intermetallic compound (IMC) layer had directional effects: the hypergravity of G+ samples effectively inhibited the formation of diffusion void defects, but this effect was weakened at 4700 *g*, as shown in Figure 22 [147]. It was thus revealed that the centrifugal force and buoyancy caused by hypergravity significantly affect the diffusion characteristics of the CuZn binary system. Centrifugal force enhances interfacial contact and promotes interfacial diffusion, while buoyancy promotes the diffusion of vacancies and atoms, resulting in the directional effects and structural behavior observed under hypergravity.

### 5.2. Structural Evolution of Alloy Materials Under Hypergravity Conditions

**Figure 23:**
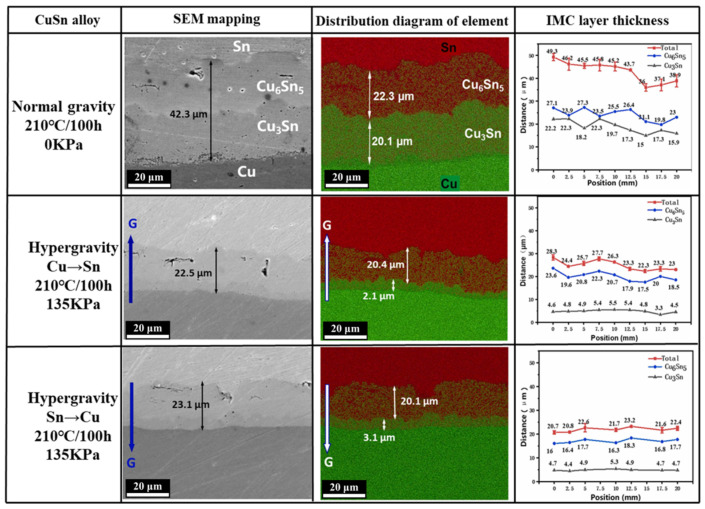
SEM and EDS maps and IMC layer thicknesses of CuSn diffusion couples treated at 210 °C for 100 h under normal/hypergravity. G represents the direction of hypergravity [149].

The evolution of material phases is closely linked to solid-phase diffusion, and hypergravity also influences the phase transformation of alloy materials, such as the formation of intermediate metal compounds. Bartek Wierzba investigated hypergravity’s impact on a material’s pressure and density. He proposed a self-consistent coupled diffusion equation to illustrate the influence of hypergravity on the two-phase growth rate of Cu_6_Sn_5_ and Cu_3_Sn in Cu-Sn diffusion couples, both theoretically and experimentally [148]. Qiao et al. [149] studied the diffusion behavior of a CuSn alloy under high temperature and hypergravity simulated by high-speed rotation and found that the growth of Cu_3_Sn in CuSn diffusion couples under hypergravity was inhibited, as shown in Figure 23. At 210 °C, hypergravity inhibits the diffusion between CuSn couples by inhibiting the formation of the intermetallic compound Cu_3_Sn and reducing the number of Kirkendall holes in Cu_3_Sn layer.

**Figure 24:**
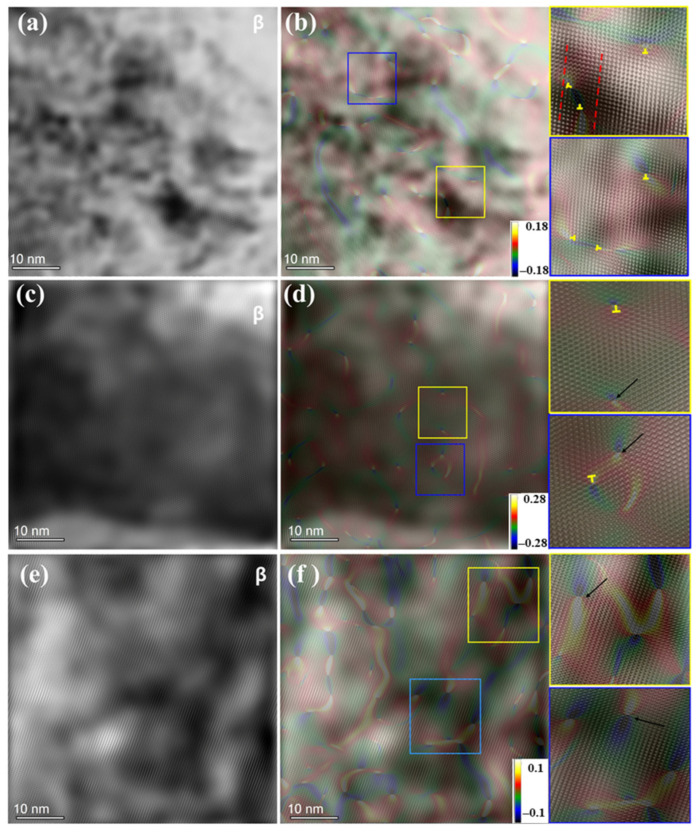
Atomic structure of β phase (CuZn) under different gravity conditions, HRTEM images, and corresponding superimposed images after filtering. (**a**,**b**) HRTEM filtered images and corresponding overlapping images of the corresponding variable distribution of β under 870 *g* hypergravity. (**c**,**d**) HRTEM filtered images and corresponding overlapping images of the corresponding variable distribution of β under 3500 *g* hypergravity. (**e**,**f**) HRTEM filtered images and corresponding overlapping images of the corresponding variable distribution of β under 4700 *g* hypergravity. The yellow marks indicate the presence of dislocations, and the black arrows indicate the presence of strain but no dislocation. The atomic strain distribution was obtained by applying the geometric phase analysis method for TEM images [147].

Simultaneously, it is essential to investigate the behavior of defects under hypergravity to complement the exploration of element diffusion mechanisms and phase evolution in solid materials. Defects present in solid crystalline materials, such as vacancies, dislocations, layer faults, twins, and grain boundaries, not only influence element diffusion and microstructure evolution but also significantly impact the mechanical properties of materials. Under normal-gravity conditions, differential diffusion rates lead to the formation of Kirkendall pores, which impede diffusion and contribute to material failure. Dislocation movement, stratification, and twin crystal formation, as well as grain boundary deformation, are critical factors influencing the atomic migration/diffusion and mechanical properties of materials. Compared to their behavior under traditional stress, the thermal diffusion and microstructure evolution of Cu-Zn diffusion couples show significant differences when subjected to hypergravity conditions. Xie et al. [147] conducted an atomic-scale mechanical study on the evolution of defect behavior under the action of hypergravity and found that the β phase, when generated under different gravity conditions, such as dislocation and other defects, decreases with an increase in hypergravity, as shown in Figure 24, especially under 4700 *g* hypergravity. The decrease in defect density represents a decrease in vacancy concentration, and the vacancy diffusion mechanism is inhibited.

The above studies fully reflect that the diffusion/movement behavior of atoms and defects (vacancy, dislocation, grain boundary) in the material will be different from the behavior under normal-gravity conditions, and the solid phase diffusion behavior and physical mechanism of the material extending from the binary alloy system to the multicomponent alloy system under hypergravity need to be further studied. It will be helpful to understand the evolution of microstructure, mechanical behavior, and properties of materials under extreme conditions.

### 5.3. The Properties of Alloy Materials Under Hypergravity Conditions

An alloy material under long-term high-speed rotating conditions is affected by the hypergravity field, which easily causes a change in the alloy structure, and the change in the alloy structure under extremely high-temperature conditions is particularly obvious after a change in the rotating device. For example, over-burnt aero-engine high-pressure turbine blades during high-speed rotation will greatly change the diffusion mode and path, change the microstructure, and even recrystallize, resulting in blade failure and engine failure [150,151,152].

**Figure 25:**
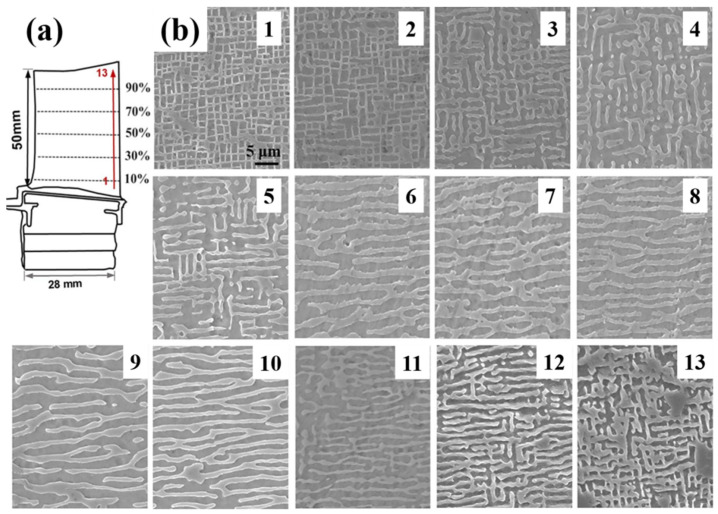
Structural evolution of turbine blades under high temperature and a hypergravity field [153]. (**a**) Schematic; (**b**) different position points. 1–13 represent a severely degraded portion from tip to stalk near the trailing edge of the blade.

For high-speed rotating systems, the traditional study of mechanical properties often uses a unified constant external force (such as creep) or different frequencies of periodic constant force (such as fatigue) loading conditions to study the effect of external forces on metals. However, due to the force transfer effect of the rigid body, the centrifugal hypergravity field has a gradient stress from the core to the outer end; that is, the magnitude of the hypergravity at different positions is different, and the structure under the coupling of temperature and stress will also be different. For typical rotating parts such as high-pressure turbine blades made of superalloys, different temperatures and external forces lead to different structural changes. Figure 25 shows that single-temperature action can only make the microstructure of superalloys coarse, and under coupled thermo-mechanical conditions, the structural rafting structure changes and ultimately changes the mechanical properties [153].

A material’s properties, such as hardness and strength, are strongly associated with the microstructure of that material. Therefore, the effects of hypergravity on material properties should be quantitatively assessed based on physical property parameters and the corresponding microstructure morphology of the materials through mechanical property measurements and corresponding microstructural analyses (e.g., SEM, TEM, EBSD). And these results can then be compared with those obtained under normal-gravity conditions. Among these analyses, the examination of the material’s microstructure and the corresponding distribution of defects such as dislocations has proven to be the most effective, as conventional material strengthening mechanisms are closely related to the interaction between microstructure and defects such as dislocations.

In the previous study mentioned, there was a lack of high-temperature and hypergravity environment equipment to dynamically simulate the extreme environment under which the service conditions of alloy materials could be assessed in situ. Moreover, most characterization studies have been conducted only at the micron scale, and the analytical problems have also been limited by the diffusion of elements and the growth of corresponding intermediate metal compounds. The lack of research on basic problems related to hypergravity leads to an insufficient understanding of the influence of hypergravity. As a result, in the field of applied research, the influence mechanism of hypergravity on the microstructure evolution, deformation and failure mechanism, and creep properties of nickel-based single-crystal superalloy materials in service remains unclear, and the correlation between microstructure evolution and properties and material damage evaluation methods under hypergravity environment is also ignored.

**Figure 26:**
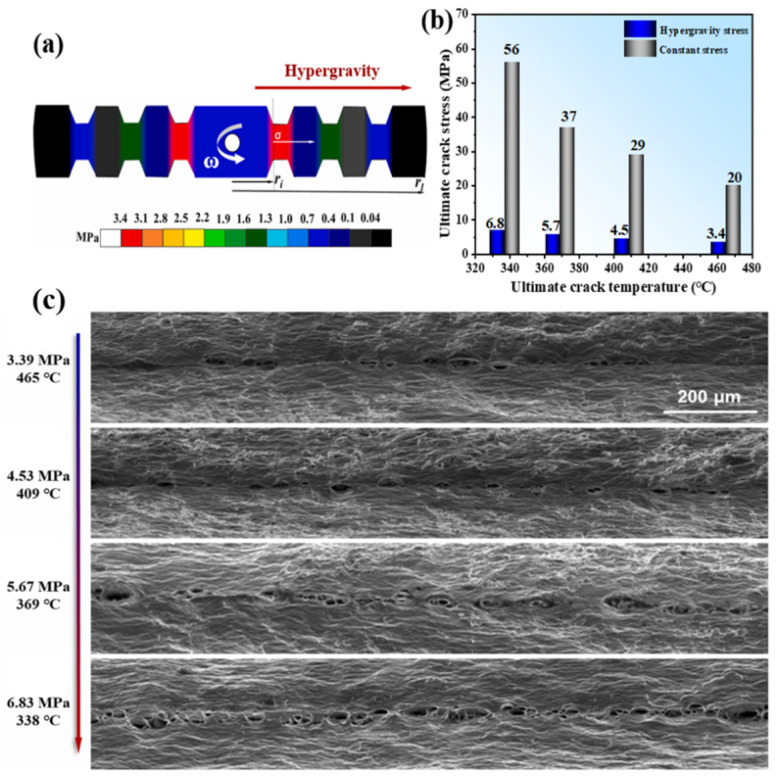
Fracture failure behavior of 1060 aluminum alloy under different hypergravity conditions [154]. (**a**) The stress of the specimen; (**b**) difference in crack limit tension under constant stress and hypergravity gradient stress; (**c**) SEM fracture morphology under different hypergravity magnitude and temperature conditions.

Based on this, some researchers have used dog bone samples to study the failure behavior of gradient hypergravity on 1060 pure aluminum. By comparing the constant force and the hypergravity force, as shown in Figure 26 [154], it has been observed that crack morphology changes under hypergravity conditions. The impact of hypergravity on crack growth is more significant than that of a constant force. At different gravity levels, the crack tip exhibits plastic deformation, and the size of the pit diminishes as gravity increases. As hypergravity intensifies, both the crack area and the number of pits increase. At the same temperature, the stress required for the sample to fracture under normal-gravity conditions and constant stress is 5–10 times that in a hypergravity field, and with an increase in the level of hypergravity, the fracture temperature of 1060 pure aluminum also decreases.

**Figure 27:**
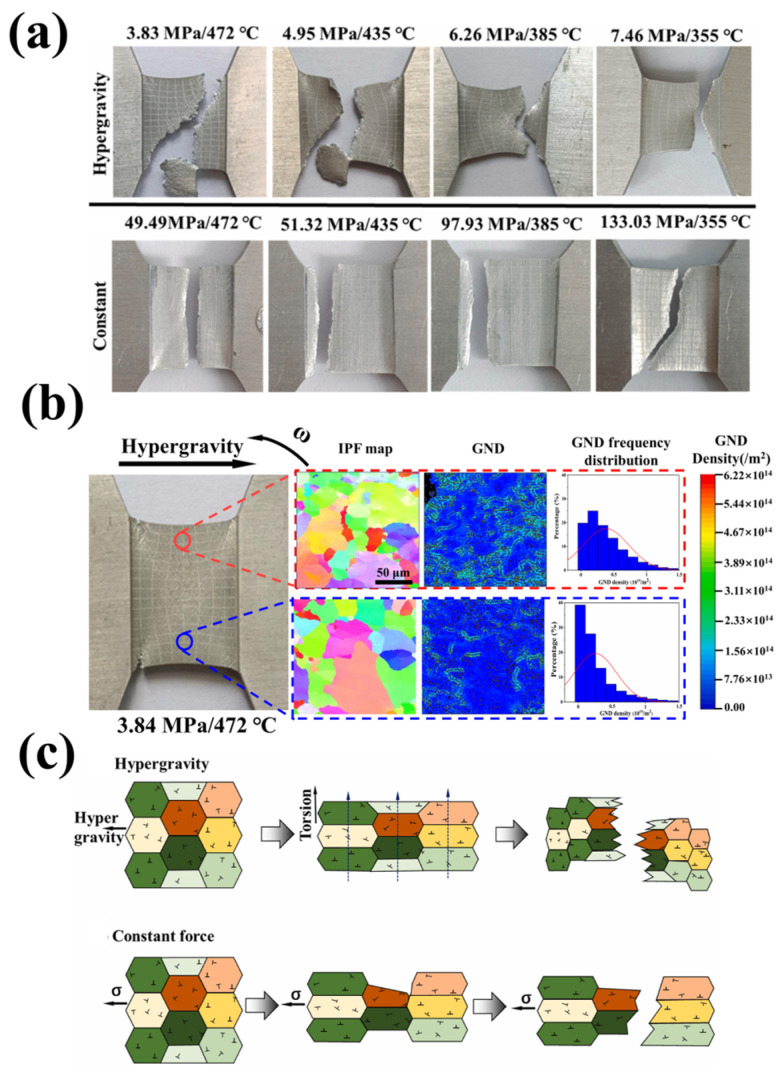
Fracture failure behavior of 7075 aluminum alloy under different hypergravity conditions [155]. (**a**) Comparison of crack tips at different gravity levels; (**b**) EBSD analysis of different crack propagation locations (IPF diagram, GND diagram and dislocation frequency distribution of the location in the dotted box, red dotted box is the upper edge of the sample, blue dotted box is the lower edge of the sample); (**c**) Schematic diagram of crack patterns under hypergravity/conventional uniaxial stress (the dotted lines are in the direction of torsion).

Similarly, the breaking strength of 7075 series aluminum alloy under normal-gravity conditions is about 10–18 times greater than that under hypergravity at the same temperature, and the differential mechanism of the cracking of alloy materials under different gravity levels/stress conditions was discussed [155], as shown in Figure 27. Under hypergravity conditions, the specimen is affected by the coupling of normal hypergravity and tangential torsional force. The torsional force splits the extended grain along the vertical direction, weakens the grain boundary, and accelerates the grain thinning at the same time. According to a geometrically necessary dislocation (GND) analysis using the electron backscattered diffraction (EBSD) method, this causes a large number of dislocations to cluster at the upper end, resulting in the vertical crack shape changing into a zigzag crack.

The above results demonstrate that hypergravity affects the evolution of a material’s structure and its properties. Specifically, for solid alloy materials subjected to a hypergravity environment, dislocations within the microstructure tend to migrate towards regions of lower hypergravity, thereby accelerating the material’s deformation and failure processes. However, the mechanism of its effect still needs to be combined with in situ mechanical transmission electron microanalysis technology to further study the influence mechanism of hypergravity on the movement of defects in materials, the crystal deformation mechanism, the initiation and expansion of microstructure defects, and material instability. In particular, the atomic-scale mechanism of the structure and property evolution of alloy materials under the coupled environment of high temperature and hypergravity has been expounded, and the similarities and differences between the effects of hypergravity and normal gravity on alloy materials have been revealed.

It is worth noting that there are some limitations in the analysis of mechanical behavior in the processes involved in hypergravity services by finite element simulation. For example, the mechanical behavior of materials in hypergravity environments involves the coupling of several physical processes (such as temperature–stress coupling), and the complexity of these processes makes it difficult to establish and solve the model accurately.

## 6. Conclusions and Perspective

This paper explores the fundamental principles and technologies associated with hypergravity conditions, liquid-phase reaction synthesis and separation, alloy metallurgy processes, and the structural and property characteristics of alloy materials. Specifically, it encompasses a comprehensive examination of hypergravity conditions, including their definitions, characteristics, and the interphase interactions and mass transfer phenomena that occur under these conditions. The research delves into the application of hypergravity techniques, such as rotating packed beds and centrifuge technologies, to enhance the efficiency of material synthesis and separation processes in both liquid and solid phases. And we also elaborate on the dynamic behavior of fluids in hypergravity environments; the mechanisms underlying liquid-phase reaction synthesis; material separation techniques; and the impact of hypergravity on various aspects of alloy metallurgy, including convection, segregation, grain refinement, dendrite texture, and the formation of gradient structures. Additionally, we also review the effects of hypergravity on solid-state diffusion, the evolution of microstructures, and the analysis of performance and failure behavior in solid-phase alloy materials.

While significant progress has been made in materials science research under hypergravity conditions, numerous scientific and engineering challenges remain to be addressed due to the complex interactions of multiphase changes in these environments.

For instance, accurately describing interphase interactions and mass transfer in hypergravity requires a comprehensive understanding and quantification of the relationships between different phases and the mass transfer processes occurring under such conditions. Further research is necessary to explore how hypergravity technology enhances the efficiency of material synthesis and separation, including its effects on reaction rates, separation efficiency, and product quality, as well as the fundamental physical and chemical mechanisms involved. Moreover, precise modeling and forecasting of hydrodynamic properties in hypergravity environments is essential. Simulation methods such as finite element analysis should develop more accurate three-dimensional models in order to explore the effects of hypergravity on the microstructure of polycrystalline materials. In metallurgy, it is vital to further elucidate the specific mechanisms by which hypergravity affects alloy metallurgy. Studies on the influence of hypergravity on solid alloy materials should concentrate on the atomic-scale mechanisms and principles that govern changes in the properties of alloy materials under hypergravity, including the modulation of material structure and properties under hypergravity conditions, which has not been explored. Ultra-high-gravity equipment (such as >100,000× g) is limited by high construction and maintenance costs, and relatively little research has been conducted on the evolution of structural and mechanical properties in complex material systems. These topics will be addressed in future research endeavors.

## Abbreviations

The following abbreviations are used in this manuscript:

CFD	Computational fluid dynamics
DFT	Density functional theory
EDS	Energy dispersive spectrometer
EBSD	Electron back scatter diffraction
GND	Geometrically necessary dislocation
HRTEM	High-resolution transmission electron microscope
IMC	Intermetallic compound
IPF	Inverse pole figure
PDAS	Primary dendritic arm spacing
RPB	Rotating packed bed
SEM	Scanning electron microscope
STR	Stirred tank reactor
TEM	Transmission electron microscope
WPCB	Waste printed circuit boards
